# Supplementary material for: Community Mobility and COVID-19 Dynamics in Jakarta, Indonesia
Source: Int J Environ Res Public Health. 2022 May 30;19(11):6671. doi: 10.3390/ijerph19116671 (PMC9180360; doi:10.3390/ijerph19116671)
Supplement: Supplementary file 1 [file ijerph-19-06671-s001.zip › ijerph-1696397-supplementary.pdf]

Table S1. Result of cross correlation function between mobility variables and daily confirmed case (in 7-day moving average).

Lag	Retail & Recreation	Grocery & Pharmacy	Parks	Transit stations	Workplaces
−21	0.4376	0.3439	0.6499	0.5653	0.2964
−20	0.4475	0.3533	0.6465	0.5763	0.3052
−19	0.4651	0.3708	0.6509	0.5883	0.3052
−18	0.4800	0.3823	0.6505	0.5989	0.3107
−17	0.4983	0.4041	0.6506	0.6158	0.3322
−16	0.5057	0.4058	0.6450	0.6237	0.3407
−15	0.5142	0.4133	0.6400	0.6329	0.3487
−14	0.5215	0.4150	0.6334	0.6411	0.3569
−13	0.5307	0.4272	0.6286	0.6500	0.3634
−12	0.5440	0.4365	0.6297	0.6579	0.3595
−11	0.5559	0.4433	0.6255	0.6657	0.3599
−10	0.5649	0.4494	0.6165	0.6763	0.3695
−9	0.5744	0.4560	0.6078	0.6861	0.3783
−8	0.5832	0.4582	0.6011	0.6947	0.3867
−7	0.5939	0.4654	0.5953	0.7036	0.3958
−6	0.6048	0.4759	0.5876	0.7136	0.4047
−5	0.6210	0.4882	0.5868	0.7223	0.4032
−4	0.6347	0.4952	0.5810	0.7306	0.4054
−3	0.6442	0.5009	0.5718	0.7407	0.4137
−2	0.6539	0.5108	0.5631	0.7504	0.4218
−1	0.6686	0.5272	0.5584	0.7654	0.4433
0	0.6764	0.5333	0.5497	0.7729	0.4487
1	0.6773	0.5339	0.5451	0.7728	0.4520
2	0.6827	0.5418	0.5421	0.7700	0.4461
3	0.6866	0.5473	0.5366	0.7677	0.4438
4	0.6878	0.5507	0.5288	0.7710	0.4578
5	0.6873	0.5515	0.5237	0.7699	0.4643
6	0.6856	0.5501	0.5190	0.7656	0.4643
7	0.6849	0.5501	0.5136	0.7619	0.4648
8	0.6833	0.5483	0.5072	0.7579	0.4650
9	0.6844	0.5502	0.5037	0.7501	0.4552
10	0.6843	0.5481	0.4963	0.7434	0.4486
11	0.6830	0.5456	0.4846	0.7406	0.4492
12	0.6820	0.5439	0.4737	0.7375	0.4478
13	0.6813	0.5416	0.4639	0.7354	0.4467
14	0.6811	0.5410	0.4532	0.7337	0.4445
15	0.6828	0.5433	0.4441	0.7337	0.4441
16	0.6855	0.5471	0.4334	0.7293	0.4356

17	0.6866	0.5465	0.4214	0.7254	0.4307
18	0.6866	0.5476	0.4080	0.7249	0.4307
19	0.6880	0.5494	0.3974	0.7243	0.4324
20	0.6888	0.5528	0.3848	0.7285	0.4456
21	0.6892	0.5540	0.3746	0.7276	0.4479
